# INTERACTIONS OF SOCIOECONOMIC AND ETHNORACIAL FACTORS SHAPING FORMAL AND INFORMAL VOLUNTEERISM IN LATER LIFE

**DOI:** 10.1093/geroni/igae098.2887

**Published:** 2024-12-31

**Authors:** Patrick Ho Lam Lai

**Affiliations:** Boston College, Chestnut Hill, Massachusetts, United States

## Abstract

Building upon previous research that identifies socioeconomic status (SES) as a pivotal factor in explaining lower volunteer rates among ethnoracial minorities in later life, this study extends our understanding by examining the interactions between SES and ethnoracial identities on their formal and informal volunteerism. By doing so, the research informs aging service practitioners and policymakers about the distinct responsiveness of different ethnoracial groups to their educational and family income levels and how these, in turn, relate to their propensity for volunteer work. By utilizing 2019 and 2021 Current Population Surveys data, this research estimates bivariate probit models for formal (e.g., through organizations) and informal volunteerism (e.g., doing favors to neighbors), including moderation with education and family income. The findings reveal that older Hispanic adults have 0.907 times or older Black adults have 0.983 times the likelihood of engaging in formal volunteer activities compared to older Asian adults, with this association becoming statistically significant upon a decrement of one educational level or family income level. Besides, older Black adults show 0.928 times the odds of engaging in informal volunteerism in comparison to Asian counterparts, with this finding being statistically significant upon a decrease in one family income level. These outcomes suggest that Asian older adults exhibit greater sensitivity to income and educational variations associated with formal and informal volunteer engagements. This research emphasizes the need to understand cultural sensitivity for lower volunteerism among older Asian adults for more inclusive aging services and policies.

